# Persistent Gastrointestinal Bleeding after Aortic Valve Replacement in Heyde’s Syndrome

**DOI:** 10.3390/jcm13154515

**Published:** 2024-08-02

**Authors:** Alexandr Ceasovschih, Raluca-Elena Alexa, Victorița Șorodoc, Anastasia Balta, Mihai Constantin, Adorata Elena Coman, Ovidiu Rusalim Petriș, Cristian Stătescu, Radu A. Sascău, Viviana Onofrei, Alexandra-Diana Diaconu, Bianca Codrina Morărașu, Gabriela Rusu-Zota, Laurențiu Șorodoc

**Affiliations:** 1 Faculty of Medicine, “Grigore T. Popa” University of Medicine and Pharmacy, 700115 Iasi, Romania; alexandr.ceasovschih@yahoo.com (A.C.); mihaiconstantin89@yahoo.com (M.C.); ado_coman@yahoo.com (A.E.C.); ovidiupetris@yahoo.com (O.R.P.); cstatescu@gmail.com (C.S.); radu.sascau@gmail.com (R.A.S.); onofreiviviana@gmail.com (V.O.); alexandra-diana_diaconu@email.umfiasi.ro (A.-D.D.); codrina.morarasu@umfiasi.ro (B.C.M.); gabrielarusu1105@gmail.com (G.R.-Z.); laurentiu.sorodoc@umfiasi.ro (L.Ș.); 2 Second Internal Medicine Clinic, Sf. Spiridon Clinical Emergency Hospital, 700111 Iasi, Romania; alexaralucaelena@gmail.com (R.-E.A.); anastasiabalta7@gmail.com (A.B.); 3 Department of Cardiology, “Prof. Dr. George I.M. Georgescu” Cardiovascular Diseases Institute, 700503 Iasi, Romania; 4 Department of Cardiology, Sf. Spiridon Clinical Emergency Hospital, 700111 Iasi, Romania; 5 Department of Pharmacology, “Grigore T. Popa” University of Medicine and Pharmacy, 700115 Iasi, Romania

**Keywords:** Heyde’s syndrome, aortic stenosis, angiodysplasias, gastrointestinal bleeding, acquired von Willebrand disease, anemia

## Abstract

Heyde’s syndrome (HS) represents an association between aortic stenosis and intestinal angiodysplasias, and it has been demonstrated that acquired von Willebrand disease plays a pivotal role in the pathophysiology of this syndrome. In patients with HS, von Willebrand factor deficiency represents an additional risk factor, further contributing to the risk of bleeding and anemia. We present the case of an 86-year-old patient diagnosed with HS and von Willebrand deficiency in 2018. Four years prior, the patient underwent surgical aortic valve replacement. Since then, she has been receiving chronic oral anticoagulation therapy with a vitamin K antagonist. The patient was admitted to the Internal Medicine Clinic due to semi-solid dark stools, diffuse abdominal pain, and asthenia. Upon examination, the patient presented with an altered general status and clinical signs suggestive of anemia. Laboratory findings revealed anemia with elevated INR and aPTT values. Colonic angiodysplasias were identified during a colonoscopy, although no sources of active bleeding were detected. On the 9th day of hospitalization, the patient experienced an episode of lower gastrointestinal bleeding. The pharmacological management was adjusted, and argon plasma coagulation was recommended. Following treatment of the angiodysplastic lesions, the patient’s clinical evolution was favorable, with the correction of the anemia.

## 1. Introduction

Heyde’s syndrome (HS) is characterized by an association between aortic stenosis (AS), intestinal angiodysplasias (ADs), and acquired von Willebrand disease (vWD) [1,2]. First reported in 1958 by Edward Heyde, it was noted that patients with calcific AS often experienced gastrointestinal (GI) bleeding, later linked to intestinal ADs. Subsequent research in 1992 proposed acquired von Willebrand syndrome as the connecting factor [1,3,4,5].

The pathophysiology of HS involves several hypotheses. AS induces ischemia and hypoperfusion, triggering angiogenesis by releasing vascular endothelial growth factor (VEGF) into the circulation. Increased shear stress through AS alters the structure of the von Willebrand factor (vWF) and contributes to the formation of intestinal ADs through various mechanisms [1,6].

**Figure 1:**
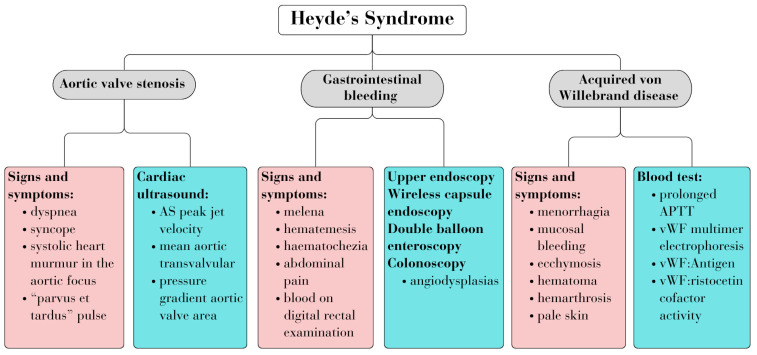
Heyde’s syndrome diagnosis.

Diagnosing HS entails identifying associated signs and symptoms of the three diseases, along with relevant biological and imaging investigations. Figure 1 summarizes the common criteria used for diagnosis (Figure 1) [4,7].

We report a case of a patient with persistent GI bleeding due to ADs following surgical correction of the AS.

## 2. Case Presentation

An 86-year-old woman was admitted to the Internal Medicine Clinic with a wide range of symptoms, including semi-solid dark stools, diffuse abdominal pain, and fatigue. These symptoms had worsened progressively over the three weeks prior to admission.

The patient’s medical history encompassed significant cardiovascular disorders (grade 3 hypertension, chronic coronary syndrome, heart failure with reduced ejection fraction (30%), supraventricular arrhythmia, moderate AS, and moderate tricuspid regurgitation) along with a prior cholecystectomy. Notably, 5 years prior to admission, the patient was diagnosed with HS, characterized by severe AS, intestinal ADs, and acquired vWD. Subsequently, a year later, she underwent surgical correction of the AS with the implantation of a mechanical valve.

At the time of admission, the patient was receiving treatment with sucrosomial iron, alpha–beta blockers, calcium channel blockers, loop diuretics, mineralocorticoid receptor antagonists, nitrates, statins, and an oral anticoagulant, specifically, Acenocoumarol, due to the implanted prosthetic aortic valve.

Upon initial clinical examination, the patient presented with an altered general state and pale, dry, dehydrated skin with decreased skin turgor. While her weight was within the normal range, her body mass index was at the lower limit (18.6 kg/m^2^). During the cardiovascular system examination, the mechanical aortic opening flap could be heard, with hemodynamic measurements showing a blood pressure of 170/62 mmHg and a heart rate of 103 bpm. Abdominal palpation elicited diffuse pain, particularly in the epigastrium, and a dark-colored stool was detected during a digital rectal examination (DRE).

The biological assessment confirmed the presence of an anemic syndrome (Hb = 7.5 g/dL), with iron and ferritin levels within normal ranges. The international normalized ratio (INR) was notably elevated at 8.72, accompanied by an increased activated partial thromboplastin time (aPTT). Throughout hospitalization, the patient exhibited fluctuating INR values ranging from 1 to 10.9, posing a considerable challenge in maintaining the target INR range of 2.5–3.5, especially considering the type of valve and its thrombogenicity.

During hospitalization, the cardiovascular risk factors were assessed. The patient had a well-controlled glycemic profile, with blood glucose and glycated hemoglobin within normal ranges. LDL-cholesterol was 69 mg/dL, with the target LDL-cholesterol being <55 mg/dL due to the patient’s very high cardiovascular risk (SCORE2OP = 58%). An evaluation of the patient’s renal function revealed a serum creatinine level of 0.99 mg/dL, corresponding to an estimated glomerular filtration rate of 51.9 mL/min/1.73 m^2^ and a normal electrolyte panel. Additionally, thyroid function tests yielded results within normal ranges. Given the patient’s abdominal pain, liver transaminases, cholestasis markers, and pancreatic enzymes were measured, all showing values within normal limits (Table 1).

**Figure 2:**
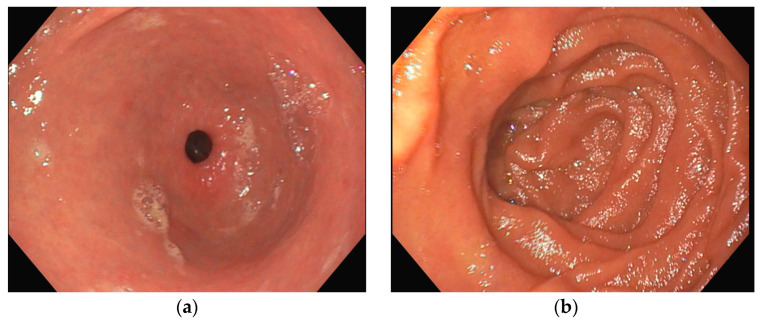
Esophagogastroduodenoscopy (**a**) antrum with pyloric sphincter; (**b**) duodenal bulb and proximal duodenum; in both images, no active bleeding lesions were identified.

**Figure 3:**
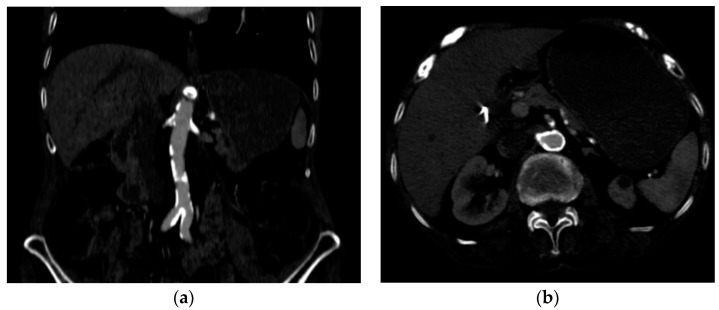
Computed tomography angiography (**a**) calcified atherosclerotic lesions on the aortic and iliac arteries; (**b**) circumferential, calcified lesions at the origin of the celiac trunk and superior mesenteric artery.

**Figure 4:**
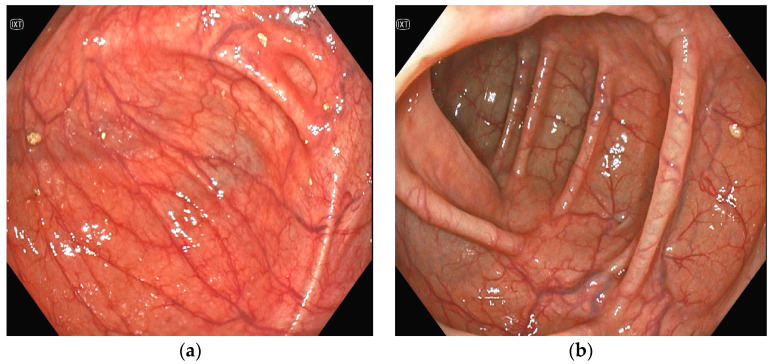
(**a**,**b**)Colonoscopy: multiple angiodysplasias without active bleeding.

Several investigations were conducted to determine the cause of anemia and abdominal pain. An esophagogastroduodenoscopy carried out in the Emergency Department did not reveal any mucosal injuries or active bleeding (Figure 2). Computed tomography angiography unveiled calcified atherosclerosis in the aorto-iliac region (Figure 3a), along with stenosis at the upper mesenteric artery and celiac trunk, though patency was maintained (Figure 3b). A colonoscopy identified ADs without active bleeding at the time of examination (Figure 4).

Therefore, we conducted a differential diagnosis for lower GI bleeding, considering several potential causes. A DRE ruled out the presence of fresh blood and hemorrhoids, and a colonoscopy did not reveal any diverticula. Acute ischemia was considered but ruled out by a CT angiography, which showed hemodynamically insignificant stenoses in the superior mesenteric artery and celiac trunk. Infectious and inflammatory causes were also ruled out, as the biological workup showed no elevated inflammatory markers, and the colonoscopy revealed no signs suggestive of an inflammatory pathology. Upper and lower endoscopies and normal tumoral marker levels excluded neoplastic causes as well. Subsequently, the patient was referred for a gynecological consultation to rule out genital bleeding. Given the patient’s history of cardiovascular diseases, a comprehensive cardiovascular assessment was performed, including an electrocardiogram (ECG), echocardiography, and a complete blood panel (Table 1). Electrocardiography revealed the presence of negative T waves in the inferior and precordial leads, while troponin levels remained stable upon repeated measurements. The echocardiographic evaluation revealed moderate mitral and tricuspid regurgitation, with the mechanical aortic valve demonstrating normal function (peak velocity = 1.208 m/s; mean gradient = 12.6 mmHg; Doppler Velocity Index = 0.56; Acceleration Time = 86 ms). The cardiac chambers were of normal dimensions, and there was a noted improvement in left ventricular ejection fraction, which was measured at 57%.

On the 9th day of hospitalization, the patient presented an episode of lower GI bleeding. Hemodynamically, the patient was stable, with a blood pressure of 120/65 mmHg and a heart rate of 78 bpm. A decline in hemoglobin levels by 2.5 g/dL (5.5 g/dL) compared to admission (7.5 g/dL) was shown, accompanied by an INR of 6.41. Consequently, two units of packed red blood cells were administrated, resulting in a subsequent increase in hemoglobin to 8 g/dL after 24 h.

Treatment strategies during hospitalization and upon discharge were aimed at improving the patient’s clinical condition, mitigating thromboembolic risks, and addressing the anemia through a comprehensive approach encompassing non-pharmacological, pharmacological, and interventional modalities.

Non-pharmacological interventions consisted of individualized dietary modifications and a tailored physical exercise regimen based on the patient’s capacity. Given the anticoagulant therapy, precautions were advised to minimize the risk of bleeding events, including avoidance of invasive procedures and foods known to interfere with the anticoagulant efficacy.

Pharmacological management during hospitalization involved a personalized therapeutic regimen, including angiotensin-converting enzyme inhibitors, calcium channel blockers, alpha–beta blockers, mineralocorticoid receptor antagonists, statins, and initiation of sodium/glucose co-transporter 2 inhibitors. Anticoagulant dosages were adjusted based on daily INR monitoring.

Upon discharge, the patient underwent interventional treatment for intestinal ADs, including argon plasma coagulation of the lesions, with a subsequently favorable evolution without digestive bleeding.

Short-term prognosis appeared favorable owing to the intervention, with hemoglobin stabilization and improved INR control with oral anticoagulant therapy. However, the long-term prognosis remains guarded due to the patient’s advanced age and the presence of multiple comorbidities.

## 3. Epidemiology

The precise prevalence of HS remains unknown due to the fact that it frequently goes underdiagnosed. Nonetheless, it is pertinent to note that AS affects approximately 7–7.5% of the general population, with GI bleeding documented in 1–3% of these individuals. Since degenerative AS has an increased incidence in the elderly, HS is frequently encountered in patients over 65 years of age [4,8].

Tjahjadi et al. conducted a study comparing the prevalence of anemia among adults who underwent aortic valve replacement (AVR) for AS versus coronary artery bypass graft. The study found that the prevalence of anemia was significantly higher in patients with AS, suggesting that HS may have a higher prevalence among this group of patients [9].

The prevalence of vWD in patients with AS is high, with approximately 79% of people with AS having low levels of vWF [10]. Additionally, the prevalence of ADs in the elderly is approximately 6% [3].

## 4. Pathophysiology

There are different hypotheses that could explain HS, suggesting that it may result from age-related degenerative processes, genetic predisposition, and dysfunction of vWF [11,12].

AS, a degenerative condition affecting the aortic valve’s structure and function, serves as a cornerstone in the pathophysiology of HS. Underlying conditions such as autoimmune disorders or metabolic diseases lead to calcification, which then narrows the aortic valve, resulting in high shear stress and low cardiac output. Hypoxia and shear stress are the main triggers for GI bleeding [10,13].

Shear stress through aortic valve stenosis leads to hypoxia and conformational changes in vWF, modifications that contribute to the formation of ADs. AS alters splanchnic blood flow, leading to low blood pressure in the superior mesenteric artery. This hemodynamic disturbance, combined with intestinal intramural pressure, fosters the formation of ADs [11,12].

As far as genetic predisposition is concerned, deficiencies in collagen type IV and a bicuspid aortic valve may play a role in the pathophysiology of HS by inducing premature calcification of the aortic valve [11].

The vWF is a multimer with a globular conformation, which, under the influence of the increased shear stress that is caused by AS, undergoes conformational changes, exposing cleavage sites susceptible to proteolysis by a metalloproteinase, ADAMTS13. As a result, smaller-sized and less hemostatically active monomers are released into circulation, thus conferring a procoagulant status, as in acquired von Willebrand syndrome type 2A [5,10,14,15,16,17].

**Figure 5:**
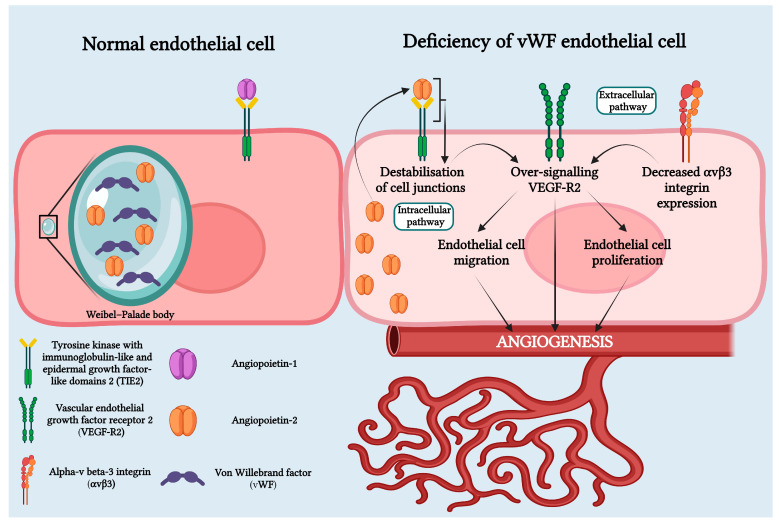
Von Willebrand deficiency in HS pathophysiology involves both intracellular and extracellular pathways. In the intracellular pathway, Weibel–Palade bodies, organelles that store vWF and Ang-2 in normal endothelial cells, are affected. In cases of vWF deficiency, Weibel–Palade bodies fail to form, causing Ang-2 to bind to the Tie-2 receptor. This leads to the destabilization of cell junctions and over-signaling of VEGF-R2. In the extracellular pathway, vWF deficiency results in decreased expression of avb3 integrin, leading to over-signaling of VEGF-R2. Over-signaling of VEGF-R2 promotes angiogenesis through endothelial cell migration and proliferation. Created with BioRender.com.

Recent studies have revealed that vWF plays an important role in angiogenesis through both intracellular and extracellular pathways. Physiologically, through the extracellular pathway, vWF inhibits angiogenesis by suppressing the signaling pathway mediated by VEGFR2. On the intracellular pathway, vWF controls angiogenesis by inhibiting Angiopoietin-2 (Ang-2), a regulatory protein of angiogenesis. This decrease in vWF results in increased release of Ang-2 from the Weibel–Palade bodies. Ang-2 bound to the TIE-1 receptor amplifies signaling through the VEGF-R2 pathway and stimulates neo-angiogenesis. Additionally, chronic hypoxia leads to high levels of VEGF that induce angiogenesis (Figure 5) [10,12,14,18,19,20].

The imbalance occurring during the process of angiogenesis can lead to blood vessel formation, which may be dysfunctional, thereby contributing to occult bleeding. This represents one of the most common causes of anemia in adults [14,18,21].

The anemia encountered in patients with AS may be multifactorial (hemorrhagic, inflammatory, due to nutritional deficiencies), but notably, these patients demonstrate a remarkable tolerance to low hemoglobin levels, attributed to the concurrent presence of diminished cardiac output, characteristic of AS [1].

In a minority of patients with HS, in whom the presence of acquired vWD has not been demonstrated, the mechanisms through which intestinal AD could occur in the presence of AS may be represented by hypoxia-induced by low cardiac output, cholesterol emboli causing hypoxia in the intestinal mucosa, and anomalies of the connective tissue. All these mechanisms may lead to the development of intestinal AD [22].

## 5. Risk Factors

A review conducted by Saha et al. attributed female sex, advanced age, and comorbidities such as hypertension, heart failure, atrial fibrillation, chronic coronary artery disease, advanced chronic kidney disease, and type 2 diabetes as risk factors for HS. The pathophysiological substrate is represented by the atherogenic process, which becomes symptomatic starting at the age of 60 and clinically manifests thereafter [22].

## 6. Diagnosis

### 6.1. Clinical Diagnosis

In HS, the hypothesis is often drawn from the patient’s history and clinical examination. The main signs and symptoms encountered in HS are depicted in Figure 1 [4]. The clinical presentation varies from occult bleeding to severe bleeding, with most patients experiencing recurrent and intermittent bleeding, along with symptoms of anemia and signs and symptoms of AS [12].

Patients with AS typically present symptoms of low cardiac output, including dyspnea, syncope, fatigue, and chest pain. On physical examination, patients often exhibit a systolic murmur in the second intercostal space and the absence of a split S2. vWD manifests with easy bruising and mucosal bleeding. The presence of ADs is suggested by GI bleeding, such as melena or blood on a DRE [4].

### 6.2. Diagnostic Investigations

All patients with suspected HS should be evaluated extensively. For these patients, the initial workup should include a complete blood count, a metabolic and coagulation panel, a fecal occult blood test, and an ECG [12]. Echocardiography is used to assess and quantify the severity of AS. The main parameters used are mean gradient, peak velocity (Vmax), and aortic valve area (AVA). Severe AS with a high gradient is established if the mean gradient is more than 40 mmHg, Vmax is ≥4 m/s, and AVA is ≤1 cm^2^. In the case of low-flow, low-gradient AS, AVA is ≤1 cm^2^, but the mean gradient is below 40 mmHg, and Vmax is less than 4 m/s; in this case, it is recommended to quantify the left ventricle ejection fraction (LVEF) and stroke volume index (SVi) in order to differentiate from severe to moderate AS (in severe AS, SVi is ≤35% mL/m^2^ independent of LVEF, but in moderate AS, LVEF is ≥50% and SVi is >35% ml/m^2^) [10,23].

Upper endoscopy and colonoscopy are the primary modalities used to examine and diagnose ADs located in the superior digestive tract and colon [10,12]. Wireless capsule endoscopy and double balloon enteroscopy are alternatives to identify ADs located in the small bowel [10,11]. Double balloon enteroscopy is superior to capsule endoscopy since it is able to visualize most of the small bowel while enabling therapeutic maneuvers. In 35% of cases, endoscopic investigation dismisses ADs [11]. Saha et al. recommend using a capsule endoscopy first, followed by a double-balloon endoscopy, due to the fact that a double-balloon endoscopy is time-consuming, more invasive, and operator-dependent [22].

The most sensitive test for the assessment of von Willebrand deficiency is gel electrophoresis, but it may take up to 7–10 days. For screening, other tests used include platelet count, which is normal, APTT and bleeding time, which may be normal or mildly prolonged, and PFA-100, which is moderately prolonged. Other tests used for confirmation are vWF antigen, FVIII coagulant activity, which can be normal or mildly low, ristocetin cofactor activity (VWF:RCo), and vWF collagen binding (VWF:CB), which can be very low, VWF:RCo/VWF:Ag ratio, and VWF:CB/VWF:Ag ratio, which is <0.7 (normal value ~1). For the diagnosis of type 2A vWD, the most effective options include gel electrophoresis, PFA-100, VWF:RCo, bleeding time, and VWF:Ag [24].

PFA-100 can be used as a screening test for vWD, but in some cases, results may be inconclusive due to multiple blood transfusions or due to the deficiency being subtle [1,4,10].

In HS, AVR is an effective treatment for GI bleeding from AD and acquired vWD [5].

In a meta-analysis conducted by Goltstein et al. including 1054 patients from 33 studies, it was demonstrated that in the majority of cases, correction of the hematologic abnormalities occurred within the first 3 days after AS correction (95%) [5].

As far as GI bleeding is concerned, the same meta-analysis showed that resolution of bleeding occurred in 73% of patients after AS correction, with differences between the types of correction used [5].

## 7. Management of Heyde’s Syndrome

While there are no guidelines for the management of HS, and most recommendations come from case reports and retrospective studies, conclusive evidence suggests that cessation of GI bleeding is often achieved after AVR [4].

Medical therapy for patients with HS focuses on managing GI bleeding and vWF deficiency. In order to address GI bleeding, medications such as somatostatin or octreotide may be prescribed to reduce the pressure on the portal venous system and control the bleeding. For the management of vWF deficiency, desmopressin may be administered, as it stimulates the release of vWF from endothelial cells, while replacement of clotting factors is also an option. Hormonal therapy may reduce the need for transfusions; in this regard, low doses of estradiol or norethisterone can be proven beneficial [11,12].

The management of HS includes AVR and the management of GI bleeding. These measures are taken depending on the bleeding status. In cases of active bleeding, the priority is to control the GI bleeding first, followed by valve repair. Typically, patients with HS require a multidisciplinary team, which includes a cardiologist, a gastroenterologist, a primary care physician, and a geriatrician [4].

GI bleeding management includes initial resuscitative measures, identifying the source of bleeding, and endoscopic interventions. Resuscitative measures include intravenous fluids and blood transfusion, while medical therapy includes the administration of somatostatin, octreotide, and thalidomide. Endoscopic interventions that may be used are argon plasma coagulation, bipolar cauterization, mechanical hemostasis with endoscopic clips, injection sclerotherapy, radiofrequency ablation, and endoscopic laser photocoagulation; any of these endoscopic interventions is chosen based on AD localization and technical availability. Embolization through CT angiography is another path to GI bleeding cessation [4,10,12,24]

In patients with contraindications for cardiac surgery, an alternative for GI bleeding cessation is elective bowel resection if the bleeding site is identified [11]. In cases of severe bleeding, if endoscopic measures cannot be taken, patients require emergency bowel resection [24].

Lourdusamy et al. propose an algorithm for managing HS, suggesting treating the GI bleeding (endoscopic, surgical, interventional) and monitoring for recurrence afterward. If GI bleeding reoccurs, referring the patient to a cardiologist is recommended in order to assess the presence of indications for AVR. Moreover, if the patient meets the requirements for AVR, even in the absence of GI bleeding, replacing the aortic valve is indicated [4].

The curative option for HS is AVR, which can be performed surgically (surgical aortic valve replacement—SAVR) or through an interventional approach (transcatheter aortic valve implantation—TAVI). However, paravalvular leakage remains the main risk, subsequently causing the recurrence of GI bleeding due to the persistence of shear stress and the hematological disorder [10,11,22]. A systematic review conducted by Saha et al. revealed that rebleeding after SAVR was lower when compared to TAVI (5.3% vs. 10.5%) [22].

TAVI has a lower risk of perioperative complications compared to SAVR, which is why it is used in patients with moderate-to-high risk for SAVR. However, the two procedures have similar success rates even in low-risk patients [4,10].

In a retrospective study, approximately 40% of patients with HS experienced recurrent GI bleeding after TAVI, compared to healthy cohorts. This occurrence may be attributed to residual paravalvular leakage after TAVI. Additionally, the persistence of ADs after TAVI suggests a potential association between paravalvular leakage and GI bleeding, which may stem not only from vascular disturbance but also from the presence of coagulopathy (Table 2) [8].

## 8. Prognosis

The prognosis of HS is highly dependent on the cessation of GI bleeding following AVR. In most cases, AVR leads to a favorable prognosis as it halts GI bleeding.

AS represents the primary trigger of the pathophysiological mechanisms in HS. Surgical correction of AS reduces shear stress and hypoxia, thereby eliminating the cause of vWF conformational changes and halting the formation of ADs, thus decreasing the hemorrhagic risk induced by acquired vWD. Therefore, in most cases, surgical correction of AS represents the curative treatment for this condition. Intestinal ADs may persist, but bleeding no longer occurs, most likely due to the elimination of the prohemorrhagic component of HS, namely acquired vWD [5,18].

However, prognosis is further influenced by the presence of other comorbidities. In a small proportion of patients experiencing rebleeding, the prognosis was guarded due to persistent anemia and the need for blood transfusions [12].

There are situations where, despite surgical correction of the AS, GI bleeding persists. This could be explained either by genetic mechanisms (deficiency of type IV collagen), cardiac structural changes (aortic bicuspid valve), or by the presence of inherited genetic coagulopathies. Chronic oral anticoagulation with vitamin K antagonists represents another risk factor, as in these situations, INR values can be easily influenced by food or medication, and maintaining the INR within the therapeutic target range is challenging, with significant variations [11,22].

## 9. Conclusions

Heyde’s syndrome is a complex condition, the pathophysiological mechanisms of which have not yet been fully elucidated. Owing to multiple reported clinical cases, it has been concluded that the main treatment modality for this condition is the correction of the stenosis via surgical or interventional means, depending on the patient’s age and comorbidities. However, in a small number of cases, even after valve correction, the persistence of intestinal AD and rebleeding was reported, raising suspicions about whether acquired vWF deficiency is the sole causative factor of the additional hemorrhagic risk or whether there are more risk factors yet unknown.

## Figures and Tables

**Table 1:** Blood tests results.

Test	Value	Normal Value
**Complete blood count**		
WBC	8.750/mm^3^	4.000–10.000/mm^3^
RBC	2.560.000/mm^3^	3.800.000–5.200.000/mm^3^
Hemoglobin	7.5 g/dL	12–16 g/dL
Platelets	286.000/mm^3^	150.000–400.000/mm^3^
MCV	91.8/fL	78–96/fL
MCH	29.3/pg	27–34/pg
MCHC	31.9 g/dL	31–36 g/dL
Lymphocytes	20.7%	20–45%
Neutrophils	70%	45–80%
Eosinophils	0.8%	0–5%
Lymphocytes	1.810/mm^3^	1.000–4.000/mm^3^
Neutrophils	6.120/mm^3^	2.000–8.000/mm^3^
Eosinophils	70/mm^3^	0–500/mm^3^
**Biochemistry panel**		
HbA1c	5.3%	4.8–5.9%
Glucose	118 mg/dL	83–110 mg/dL
Total-cholesterol	125 mg/dL	120–200 mg/dL
HDL-cholesterol	38 mg/dL	40–60 mg/dL
LDL-cholesterol	69 mg/dL	10–130 mg/dL
Triglycerides	86 mg/dL	35–150 mg/dL
Uric acid	5.2 mg/dL	2.5–6.2 mg/dL
Creatinine	0.99	0.5–1.2 mg/dL
eGFR	51.9 mL/min/1.73 m^2^	>90 mL/min/1.73 m^2^
ALT	10 U/L	5–34 U/L
AST	11 U/L	2–55 U/L
Bilirubin	0.23 mg/dL	0.20–1.20 mg/dL
Alkaline phosphatase	110 U/L	40–150 U/L
Amylase	62 U/L	28–100 U/L
Lipase	39 U/L	8–78 U/L
**Coagulation panel**		
INR	8.72	0.8–1.25
APTT	114.40 s	24–35 s
Prothrombin time	95.9 s	10–14 s

**Abbreviations:** ALT = alanine transaminase; APTT = activated partial thromboplastin time; AST = aspartate aminotransferase; eGFR = estimated glomerular filtration rate HbA1c = hemoglobin A1c; INR = international normalized ratio; MCH = mean corpuscular hemoglobin; MCHC = mean corpuscular hemoglobin concentration; MCV = mean cell volume; RBCs = red blood cells; WBCs = white blood cells.

**Table 2:** GI bleeding after AVR.

Type of Study	No of Patients	Outcome	References
Case report	1	GI bleeding after 1 year from mechanical AVR. GI bleeding cessation after administration of octreotide 20 mg, once a month.	[25]
Retrospective cohort study	57	Twelve patients experienced GI bleeding after AVR, and three patients required additional blood transfusions.	[26]
Case report	1	GI bleeding from intestinal ADs 2 months after TAVI for AS.	[27]
Retrospective cohort study	47	In total, 32% of patients with HS had recurrent GI bleeding.	[28]
Case report	2	Persistent GI bleeding after SAVR and TAVI. GI bleeding cessation after bevacizumab.	[29]
Retrospective cohort study	164	In total, 34 patients out of 164 had recurrent GI bleeding after TAVI.	[30]
Case report	1	Patient with AVR and recurrent GI bleeding.	[31]
Case report	1	Massive GI bleeding from ADs 10 months after AVR.	[32]

**Abbreviations:** ADs = angiodysplasias; AS = aortic stenosis; AVR = aortic valve replacement; GI = gastrointestinal; HS = Heyde’s syndrome; SAVR = surgical aortic valve replacement; TAVI = transcatheter aortic valve implantation.
